# Structural physical activity restriction, cerebrovascular and diabetes mortality, and long-term care intensity in Japan: An ecological panel study across 47 prefectures (2013−2022) with a COVID-19 counterfactual analysis

**DOI:** 10.1016/j.dialog.2026.100320

**Published:** 2026-06-11

**Authors:** Akira Kimura

**Affiliations:** Division of Public Health, Graduate School of Health Sciences, Gunma Paz University, Takasaki, Gunma, Japan

**Keywords:** Physical activity, Cerebrovascular disease, Diabetes mellitus, Long-term care, Ecological panel, Japan

## Abstract

**Background:**

Physical inactivity is an established cardiovascular risk factor at the individual level, but the population-level mortality cost attributable to modifiable, built-environment constraints on daily physical activity — rather than to individual behaviour — has lacked an empirical benchmark, leaving investment in walkable urban design without a clear health-economic case. We asked whether structurally constrained population physical activity is associated with cause-specific mortality, and used the 2020 COVID-19 mobility restriction — a society-wide curtailment that could never be imposed experimentally — to estimate the effect size of an acute collective PA shock on cardiovascular and metabolic deaths.

**Methods:**

10-year ecological panel of Japan's 47 prefectures (2013–2022; *N* = 470). We modelled cerebrovascular (CVD), diabetes (DM), and heat-illness deaths with age-sex-standardized CRE Mundlak Poisson regression and the severe long-term-care ratio (SHR) with a fractional logit, applying stepwise confounder adjustment (M0–M5: medical resources, income, education, age structure, birth rate). 2020 excess was estimated against a counterfactual trend trained on 2013–2019.

**Results:**

Higher car-only commuting (range 8.5–79.0%) was associated with greater CVD (IRR per SD = 1.064; 95% CI 1.037–1.093; +29% across the observed range) and DM mortality (IRR = 1.030; 1.000–1.060), and with greater long-term-care severity (SHR OR = 1.39; 1.18–1.64). CVD persisted across all five adjustment models; DM attenuated to non-significance after consumption-expenditure adjustment, consistent with a correlated socioeconomic cluster rather than an independent driver. In 2020, the most car-dependent tertile recorded +8.3% excess CVD deaths (95% CI +2.8% to +14.0%) versus near-zero excess elsewhere — a quantifiable effect of acute collective PA restriction on cardiovascular mortality.

**Conclusion:**

A modifiable feature of modern life — car dependence — carries a measurable population-level cardiovascular mortality cost that amplifies when behavioural PA is acutely curtailed. The effect size justifies treating walkable urban design and public transit as primary NCD prevention in super-ageing societies, not as ancillary transport policy.

**Funding:**

None declared.

## Highlights

• Car-only commute rate (PAR) varied 8.5%–79% across Japan's 47 prefectures.
• Higher PAR linked to higher cerebrovascular mortality after age-sex standardization.
• PAR effect on CVD robust across 5 hierarchical confounder models (M0–M5).
• High-PAR prefectures had +8.3% excess CVD deaths during 2020 COVID-19 disruption.
• DM-PAR attenuated after income proxy adjustment, marking structural disadvantage.

## 1 Introduction

Physical inactivity is estimated to cause approximately 3.2 million deaths annually worldwide and is a major driver of non-communicable diseases including cardiovascular disease (CVD), type 2 diabetes mellitus (DM), and functional decline requiring long-term care [1], [2], [17], [18]. At the individual level, the inactivity–cardiovascular link is well established by cohort and randomised-trial evidence [12], [17], [18], and clinical and behavioural guidance accordingly emphasises individual physical-activity (PA) prescription. What remains less well quantified is the population-level mortality cost attributable to the built and transportation environments that determine how much daily PA is even possible: when commuting, errands, and social access are car-dependent by design, incidental walking is displaced from daily routine [3], [4], [5], [6], and the question becomes not whether individuals choose to be active but whether the environment they inhabit makes activity ordinary. The size of this environmental contribution — the effect that no individual exhortation can directly modify — is the empirical gap motivating this study, because without it, investment in walkable urban design and public transit as health policy is being argued on first principles rather than measured benefit.

**Fig. 1:**
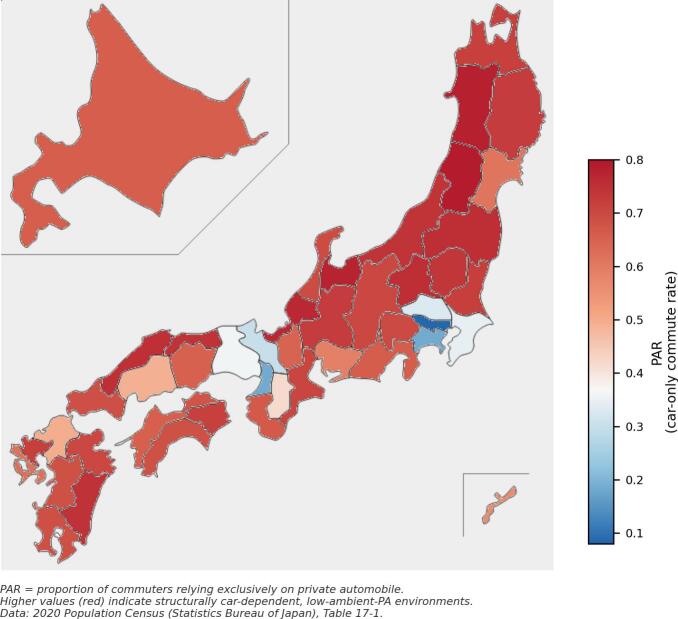
Geographic distribution of structural physical activity restriction (PAR) across 47 Japanese prefectures (2020 census). Choropleth map showing the proportion of commuters relying exclusively on automobile transportation. PAR ranges from 8.5% (Tokyo) to 79.0% (Yamagata), reflecting decades-long differences in transportation infrastructure investment between transit-oriented metropolises and car-dependent rural regions.

Japan offers an unusually clean ecological setting in which to put a number on this environmental contribution. Its 47 prefectures span a fivefold gradient in automobile commuting dependence — from 8.5% of commuters relying exclusively on cars in Tokyo to 79.0% in Yamagata (Fig. 1) — reflecting decades-long, infrastructure-level differences between densely urbanised transit-oriented metropolises and car-dependent rural regions. Because this gradient is structural rather than individually determined, it is largely exogenous to short-term individual health behaviours: prefecture-level car dependence is a feature of the place, not a sum of its residents' transport choices in any given year. At the same time, Japan's comprehensive national statistics (Vital Statistics, the Long-Term Care Insurance reports, and the national census) provide 10-year longitudinal administrative panel data for all 47 prefectures, allowing the environmental effect to be estimated against a decade of within-prefecture and between-prefecture variation rather than from a single cross-section [22], [23].

To estimate not just the chronic but also the acute population effect of restricting PA, we use the 2020 COVID-19 pandemic as a natural experiment. Nationwide containment measures abruptly curtailed commuting, outdoor activity, and gymnasium use, substantially reducing ambient daily physical activity across the entire population [13], [14], [15], [16]. No ethics board would ever approve a deliberate, society-wide curtailment of population physical activity for research purposes; the pandemic, in effect, performed such an experiment for us. Layered on pre-existing structural PA environments, this acute disruption allows a counterfactual contrast: were 2020 cardiovascular and metabolic mortality rates higher in prefectures that were already PA-restrictive than in those that were not, and by how much? The size of any 2020 excess, stratified by structural PA environment, is the most direct quantitative read-out of how much population health hangs on the ability to walk in daily life. We treat 2020 as a counterfactual-trend extrapolation rather than a formal interrupted time series or difference-in-differences design, and report the effect size with explicit uncertainty.

This study addresses three pre-specified research questions that together form a coherent assessment of the environmental contribution to population physical-activity health. (1) Does the structural physical-activity environment, measured by car-only commuting dependence (PAR), predict cause-specific mortality — specifically cerebrovascular and diabetes mortality — at the prefecture level, and is the cerebrovascular signal independent of the major correlated socioeconomic and demographic confounders, when these are introduced stepwise? (2) Does the same structural environment shape not only fatal but also disabling outcomes, indexed by the severe long-term-care ratio (SHR), with implications for the lived-disability burden of car dependence in a super-ageing society? (3) When an acute, society-wide curtailment of behavioural PA was imposed by the 2020 COVID-19 pandemic, did populations already inhabiting PA-restrictive environments experience a quantifiable excess cardiovascular mortality, and how large is that effect? Answering these in sequence is intended to convert a long-standing first-principles claim about walkable environments into an empirical effect-size benchmark on which policy decisions about urban-design investment can be evaluated.

## 2 Methods

### 2.1 Study design and setting

We conducted an ecological panel study using prefecture-level administrative data for all 47 Japanese prefectures over 10 consecutive years (2013–2022; *N* = 470 prefecture-years). The prefecture was the unit of analysis. The study exploits (a) between-prefecture variation in structural PA environments (primary analysis) and (b) the COVID-19 pandemic year 2020 as an exogenous PA-disruption period in a counterfactual-trend extrapolation analysis.

### 2.2 Data sources and variables

Table 1 summarises all study variables. Outcome data were extracted from the Vital Statistics of Japan (Ministry of Health, Labour and Welfare [MHLW], 2013–2022): annual prefecture-level all-age deaths attributed to cerebrovascular disease (ICD-10: I60–I69), diabetes mellitus (E10–E14), and heat illness (T67; validation outcome). Because prefecture-level T67 tabulations are subject to systematic national-level under-reporting relative to the official national T67 total, prefecture-year counts were multiplicatively rescaled by a year-specific factor f_t (national official total ÷ aggregate raw total) before analysis; this preserves the within-year between-prefecture distribution (see Supplementary eMethods). The rescaling and the subsequent indirect age-sex standardization (which generates the log-expected-deaths offset, described below) are applied in that order; because f_t is a year-specific multiplicative constant, the rescaling is absorbed by the year fixed effects in the CRE Mundlak specification and cannot generate spurious cross-sectional association with the time-invariant PAR. The rescaling is applied only to the T67 validation outcome, not to the CVD or DM models. Long-term care data were obtained from the Annual Report on Long-Term Care Insurance (MHLW), with the severe care ratio (SHR) defined as the proportion of LTCI-certified recipients at care levels 3–5 out of all certified recipients.

**Table 1:** Variable descriptions and descriptive statistics (*N* = 470 prefecture-years, 47 prefectures × 2013–2022).

Variable	Operational definition	Mean (SD)	Range	Source
*Outcomes*				
CVD deaths (I60–I69)	Annual prefecture-level cerebrovascular disease deaths	2293 (1755)	573–9690	Vital Statistics
DM deaths (E10–E14)	Annual diabetes mellitus deaths	295 (249)	58–1398	Vital Statistics
SHR	Severe LTCI care ratio: levels 3–5 / all certified	0.358 (0.033)	0.282–0.463	LTCI Report
T67 deaths (validation)	Heat illness deaths (adj. For reporting artefact)	21.8 (37.9)	0–371	Vital Statistics
*Exposure*				
PAR	Car-only commute rate (2020 census; structural PA proxy)	0.613 (0.176)	0.085–0.790	2020 Census
*Covariates*				
Temp anomaly (°C)	Jul–Aug mean max temp, within-prefecture deviation from 10-yr mean	0.00 (0.68)	−1.67 − +1.67	JMA AMeDAS
Energy expenditure (¥)	Annual household fuel/utilities (air-conditioning access proxy)	22,798 (2868)	15,764–33,197	SSDSE-B
Expected deaths (offset)	log(E_it), age-sex-standardized expected deaths via indirect standardization (Breslow-Day) using national age-sex-specific reference rates	–	–	e-Stat / Vital Statistics

Abbreviations: CVD, cerebrovascular disease; DM, diabetes mellitus; SHR, severe care ratio; PAR, physical activity restriction proxy; LTCI, long-term care insurance; JMA, Japan Meteorological Agency. SD, standard deviation.

The primary exposure variable — the Physical Activity Restriction proxy (PAR) — was the proportion of commuters aged ≥15 years relying exclusively on private automobiles for commuting, derived from the 2020 Population Census (Statistics Bureau of Japan, Table 17–1). PAR captures structural ambient PA opportunity in daily life: prefectures with high PAR are car-dependent with limited pedestrian infrastructure, whereas low-PAR prefectures are transit-oriented with substantially more incidental walking and cycling in daily routine. PAR was operationalised as a time-invariant structural covariate (fixed at the 2020 census value). This is theoretically and empirically justified: Japan's Transportation Infrastructure Survey and prior census rounds demonstrate that prefecture-level automobile commuting rates exhibit strong structural persistence over 5-year intervals (rank correlation ρ > 0.97 between successive census rounds), reflecting multi-decade infrastructure investment patterns that do not change rapidly. Furthermore, the COVID-19 counterfactual analysis targets 2020 as the shock year, making the 2020 census value the most temporally appropriate structural measure.

Annual mean maximum temperatures for July and August (Jul–Aug Tmax) were obtained from the Japan Meteorological Agency (JMA) Automated Meteorological Data Acquisition System (AMeDAS). Temperature was expressed as a within-prefecture anomaly (Temp_anom) — deviation of each year's Jul–Aug Tmax from that prefecture's 10-year mean — to remove time-invariant local climate differences. Saga Prefecture, lacking a dedicated AMeDAS prefectural station, was estimated as the arithmetic mean of adjacent Fukuoka and Nagasaki prefectures. Annual household energy expenditure (fuel and utilities) from the Social and Demographic Statistics by Region (SSDSE-B) was included as an air-conditioning access proxy. For count outcomes (CVD, DM, T67), the model offset was log(expected deaths E_it), where E_it was computed by indirect age-sex standardization using national age-sex-specific death rates as the reference (Breslow-Day method) [24] and prefecture × 5-year-age-group × sex × year populations from e-Stat (Statistics Bureau of Japan); this replaces the earlier log(Type-1 LTCI insured ≥65) offset, which mismatched the all-age numerator.

### 2.3 Statistical analysis

#### 2.3.1 Main model — CRE Mundlak specification

Because PAR is time-invariant at the prefecture level, its coefficient cannot be identified by conventional within-prefecture fixed-effects (FE) estimators (perfect collinearity with prefecture fixed effects). To recover between-prefecture estimates of PAR while retaining within-group control for time-varying confounders, we adopted the Mundlak (1978) [7] correlated random effects (CRE) approach. We estimated pooled Poisson pseudo-maximum likelihood (PPML) models augmented with prefecture-level group means of the time-varying covariates:

(1) $$\log\;E\left[Y\_\mathrm{it}\right]=\alpha +\beta 1\cdot \mathrm{Temp}\_\mathrm{anom}\_\mathrm{it}+\beta 2\cdot \mathrm{Temp}\_{\mathrm{anom}}^{2}\_\mathrm{it}+\beta 3\cdot \mathrm{PAR}\_i+\mathrm{\beta 4}\cdot \left(\mathrm{PAR}\times \mathrm{Temp}\_\mathrm{anom}\right)\_\mathrm{it}+\beta 5\cdot \mathrm{Energy}\_\mathrm{it}+\lambda 1\cdot ®\mathrm{Temp}\_{\mathrm{anom}}^{2}\_i+\lambda 2\cdot ®\mathrm{Energy}\_i+\gamma \_t+\log\left(E\_\mathrm{it}\right)$$

where Y_it is the outcome count; PAR_i is the time-invariant structural PA exposure; γ_t represents year fixed effects (nine year dummies, 2013 as reference); and log(E_it) is the indirect age-sex-standardized expected-deaths offset. The Mundlak correction terms λ1·̅Temp_anom^2^_i and λ2·̅Energy_i are prefecture-level group means of the respective time-varying covariates; under the conditional-mean independence assumption that the prefecture random effect, after this adjustment, is uncorrelated with PAR, the PAR coefficient can be interpreted as a between-prefecture association conditional on the included time-varying covariates [7], [8]. The correction does not eliminate confounding by unobserved time-invariant prefecture characteristics. Note that Temp_anom is defined as the within-prefecture deviation from each prefecture's 10-year mean and therefore has a prefecture-level group mean of zero by construction; the group mean of Temp_anom (linear term) was accordingly excluded from the Mundlak correction set. Temp_anom^2^, however, is a non-negative variable whose prefecture-level group mean represents within-prefecture temperature variability (a positive quantity by construction) and was retained as a Mundlak correction term. Standard errors were clustered at the prefecture level (47 clusters, CR1 sandwich estimator). PPML was fitted as quasi-Poisson to allow for overdispersion (φ̂ reported in Supplementary eTable 1). For SHR (a bounded proportion in (0,1)), we replaced the Poisson model with a Papke-Wooldridge fractional logit (quasi-binomial GLM with logit link) [19], with the Mundlak correction applied only to group means of time-varying covariates. (The originally submitted CRE OLS specification erroneously included the group mean of SHR itself as a regressor, which mechanically forced an artefactual ordinary R^2^ of approximately 0.966 and a PAR coefficient near 1.000; that misspecification has been corrected. The two reported fit statistics — the original OLS R^2^ = 0.966 and the new fractional-logit McFadden pseudo-R^2^ = 0.002 — are not directly comparable because they index different model families on different scales, but their juxtaposition makes the artefactual nature of the original explanatory power transparent.) Effect sizes for PAR are reported per 1 SD increase (SD = 0.176), per 10 percentage-point increase, and across the full observed range (0.085 to 0.790).

### 2.4 Sensitivity analysis

As a within-prefecture sensitivity check, we estimated PPML models with full prefecture and year fixed effects. Because PAR is exactly collinear with prefecture fixed effects, PAR (and the PAR × Temp_anom cross-level interaction) were dropped from the FE specification before estimation, so that the design matrix is of full column rank. Only the coefficients of the time-varying covariates (Temp_anom, Temp_anom^2^, energy expenditure) are reported in this specification (Supplementary eTable 2). Additional sensitivity analyses included (i) hierarchical adjustment for prefecture-year proxies of structural confounders — medical-facility density, consumption expenditure (income proxy), university student share (education proxy), ≥65 population share, and birth rate — added stepwise as models M0–M5 (Supplementary eTable 6, eFigure S8); (ii) leave-one-prefecture-out and leave-one-year-out refitting of the main CRE Mundlak Poisson model (eTables 7–8, eFigures S6–S7); and (iii) alternative offset specifications using total resident population and Type-1 LTCI insured ≥65 (results substantively unchanged).

### 2.5 Counterfactual analysis for 2020

We estimated a counterfactual-trend extrapolation model trained on pre-pandemic data (2013–2019; 329 prefecture-years) using the CRE Mundlak Poisson specification with the year fixed effects replaced by a linear calendar-year trend (year_c = calendar_year −2013) to enable extrapolation to 2020. Predicted values (Ŷ_i,2020) represent the expected 2020 outcome had the pre-pandemic trend continued, conditional on observed 2020 covariates (temperature anomaly, energy expenditure) and the structural PAR. Excess deaths were computed as Δ_i = Y_i,2020 − Ŷ_i,2020, with Δ_i / Ŷ_i,2020 × 100 as the excess percentage. Prefectures were stratified by PAR tertile (Low: PAR ≤ 0.566 [*n* = 16]; Mid: 0.566 < PAR ≤ 0.733 [n = 16]; High: PAR > 0.733 [*n* = 15]) and excess percentages were aggregated by tertile. Ninety-five percent confidence intervals for tertile-level excess percentages were obtained from 2000 prefecture-block bootstrap resamples of the 2013–2019 training set, with the counterfactual model refit on each replicate. Robustness to trend specification was assessed by re-fitting with (i) a quadratic year trend, (ii) a piecewise-linear trend with an optional breakpoint identified by AIC, and (iii) a prefecture-level ARIMA(1,1,0)-with-drift specification (Supplementary eTable 5, eFigure S5). A formal continuous interaction test (Year2020 × PAR) was conducted on the full 2013–2020 panel (*N* = 376 prefecture-years; 47 prefectures × 8 years); the resulting interaction IRR, 95% CI, and *p*-value are reported in the main text. We emphasise that the continuous-interaction test and the tertile-stratified counterfactual analysis address complementary but distinct quantities: the continuous test asks whether the 2020 deviation scales linearly with PAR, whereas the tertile estimate quantifies the 2020 excess concentrated in the top of the PAR distribution; the two can diverge in finite ecological samples and we report both. Multiple concurrent 2020 changes (healthcare-seeking, cause-of-death certification, rural–urban epidemic timing, economic conditions, dietary patterns, telework adoption) complicate attribution to PA restriction alone and are discussed in Limitations.

### 2.6 Ethics

This study analysed publicly available secondary ecological data and did not involve individual participants. Ethics review was not required under Japanese regulations.

### 2.7 Software

Python 3.12 (statsmodels 0.14). All code and processed data are available from the corresponding author on request.

## 3 Results

### 3.1 Descriptive statistics

The study panel comprised 470 prefecture-year observations. CVD mortality ranged from 573 to 9690 deaths per prefecture-year (mean: 2293; SD: 1755), and DM mortality from 58 to 1398 (mean: 295; SD: 249), reflecting substantial variation driven by population size and age structure. Mean SHR was 0.358 (SD: 0.033). PAR ranged from 0.085 (Tokyo) to 0.790 (Yamagata), with a national mean of 0.613. High-PAR prefectures (Yamagata 79.0%, Akita 78.0%, Toyama 78.0%, Fukui 76.0%, Gunma 75.0%) are predominantly rural with extensive road infrastructure and limited rail connectivity. Low-PAR prefectures (Tokyo 8.5%, Kanagawa 18.0%, Osaka 19.0%, Kyoto 30.0%) are densely urbanised transit hubs. Temperature anomalies ranged from −1.67 °C to +1.67 °C with a within-prefecture mean of zero by construction.

### 3.2 Main analysis: CRE Mundlak panel models

Table 2 presents the CRE Mundlak Poisson results for CVD and DM mortality, with log(age-sex-standardized expected deaths) as the offset. After Mundlak correction for the prefecture-level group means of time-varying covariates and year fixed effects, PAR was positively associated with both outcomes.

**Table 2:** CRE Mundlak Poisson model results — incidence rate ratios for cerebrovascular mortality (CVD, I60–I69) and diabetes mortality (DM, E10–E14) (*N* = 470 prefecture-years, 47 prefectures × 10 years).

Variable	CVD IRR	CVD 95% CI	CVD p	DM IRR	DM 95% CI	DM p
Temp anomaly (°C)	1.003	(0.990, 1.015)	0.684	1.013	(0.989, 1.038)	0.304
Temp anomaly^2^	0.994	(0.985, 1.004)	0.254	1.008	(0.991, 1.025)	0.364
PAR (per 1 SD, SD = 0.176)	1.064	(1.037, 1.093)	<0.001 ***	1.030	(1.000, 1.060)	0.049 *
PAR (per 10 percentage-point increase)	1.035	(1.020, 1.051)	<0.001 ***	1.017	(1.000, 1.034)	0.049 *
PAR (across observed range, 0.085 → 0.790)	1.290	(1.158, 1.436)	<0.001 ***	1.127	(1.000, 1.270)	0.049 *
PAR × Temp anomaly	0.993	(0.975, 1.011)	0.456	0.989	(0.967, 1.011)	0.319
Energy expenditure (z)	1.020	(1.007, 1.033)	0.003 **	1.041	(1.013, 1.070)	0.004 **
[M] Mean Temp anomaly^2^ per prefecture	0.789	(0.685, 0.910)	0.001 **	0.952	(0.802, 1.131)	0.577
[M] Mean energy expenditure per prefecture	1.005	(0.938, 1.077)	0.893	1.018	(0.927, 1.118)	0.713
N (prefecture-years)	470			470		
Pearson dispersion φ̂	4.21			2.86		
Log-likelihood	−11,443.3			−2038.7		
AIC	22,920.7			4111.4		

Notes: IRR = incidence rate ratio. Model: Pooled Poisson PPML (quasi-Poisson) with Mundlak (1978) correction terms and year fixed effects (2013 reference). Offset = log(age-sex-standardized expected deaths E_it), computed by indirect standardization using national age-sex-specific reference rates (Breslow-Day). SE clustered at prefecture level (47 clusters, CR1). [M] = Mundlak correction term (prefecture-level group mean of the time-varying covariate). PAR is time-invariant and identified via between-prefecture variation under the CRE conditional-mean independence assumption. PAR effect sizes are reported per 1 SD (SD = 0.176 ≈ 17.6 percentage points), per 10 percentage-point increase, and across the full observed range (0.085 → 0.790). *** p < 0.001; ** p < 0.01; * p < 0.05; † p < 0.10.

Cerebrovascular mortality (I60–I69). Prefectures with higher PAR had elevated CVD mortality (per 1 SD increase in PAR: IRR = 1.064; 95% CI: 1.037–1.093; *p* < 0.001; per 10 percentage-point increase: IRR = 1.035; across the full observed range Tokyo→Yamagata: IRR = 1.290; 95% CI: 1.158–1.436). After indirect age-sex standardization and adjustment for within-prefecture temperature anomaly, household energy expenditure, and prefecture-level Mundlak terms, this corresponds to approximately 29% higher age-sex-standardized CVD mortality in a prefecture at the maximum observed PAR versus the minimum. This association is robust to hierarchical adjustment for medical-facility density, consumption expenditure (income proxy), university student share (education proxy), ≥65 population share, and birth rate (M0–M5: IRR per 1 SD ranges 1.064–1.074, all *p* < 0.001; Supplementary eTable 6, eFigure S8). Temperature anomaly was not significantly associated with CVD mortality (IRR = 1.003; *p* = 0.684), as expected given the non-thermal pathway. Higher energy expenditure was positively associated with CVD mortality (IRR = 1.020 per SD; *p* = 0.003), likely reflecting cold-climate confounding (heating costs and higher stroke rates in northern prefectures). The Mundlak correction term for mean Temp_anom^2^ was negatively associated (IRR = 0.789; *p* = 0.001), suggesting that structurally more temperature-volatile prefectures have lower underlying CVD rates, possibly reflecting climate adaptation or dietary patterns.

Diabetes mortality (E10–E14). A positive PAR–DM association was observed in the base model (per 1 SD: IRR = 1.030; 95% CI: 1.000–1.060; *p* = 0.049; across the observed range: IRR = 1.127). This association attenuated upon hierarchical adjustment for consumption expenditure (M2: IRR/SD = 1.008, *p* = 0.650) and remained non-significant through M5 (full adjustment: IRR/SD = 1.011, *p* = 0.783; Supplementary eTable 6, eFigure S8), consistent with DM-PAR capturing a cluster of correlated structural socioeconomic conditions (low income, low education, demographic ageing) rather than an isolated independent driver. Temperature anomaly was not significantly associated with DM mortality (IRR = 1.013; *p* = 0.304), and energy expenditure was positively associated (IRR = 1.041 per SD; *p* = 0.004). The cross-level interaction between PAR and temperature anomaly was not significant in either outcome (CVD: *p* = 0.456; DM: *p* = 0.319).

Validation — Heat illness mortality (T67). As an internal validation, the heat illness model yielded a strongly significant positive association between temperature anomaly and heat deaths (IRR = 1.479 per 1 °C anomaly; 95% CI: 1.248–1.753; *p* < 0.001), confirming the model's sensitivity to temperature-attributable outcomes. PAR showed a borderline negative association (IRR = 0.405; *p* = 0.051), consistent with rural/car-dependent prefectures experiencing lower urban heat-island effects and thus lower heat mortality, converging with prior time-series evidence on COVID-19-associated changes in physical activity and heat-illness mortality in Japan [13], [14], [15], [16].

### 3.3 Severe long-term care ratio (SHR)

Table 3 presents the Papke-Wooldridge fractional logit results19 for the severe LTCI care ratio, replacing the originally submitted CRE OLS specification (which had erroneously included the outcome's own group mean as a Mundlak correction term, producing an artefactual ordinary R^2^ of 0.966 with a PAR coefficient mechanically forced near 1.000). With the correctly specified fractional logit (Mundlak correction applied only to time-varying covariates), PAR was positively associated with SHR (OR per unit PAR = 1.392; 95% CI: 1.180–1.642; *p* < 0.001; average partial effect on the proportion scale = 0.076; McFadden pseudo-R^2^ = 0.002). Note that the previous R^2^ (OLS, 0.966) and the new pseudo-R^2^ (McFadden, fractional logit, 0.002) are not directly comparable: they index different model families on different scales, and we present both only to make explicit that the original headline R^2^ was an artefact of the misspecification and not a substantive measure of fit. A beta regression sensitivity specification yielded a consistent positive PAR coefficient (β = 0.331, SE = 0.084). The within-prefecture temperature anomaly did not reach significance; the quadratic temperature term was not significant (β = −0.0162; *p* = 0.259).

**Table 3:** Papke-Wooldridge fractional logit results — severe long-term care ratio (SHR) on the (0,1) proportion scale (*N* = 470 prefecture-years).

Variable	β (logit)	SE (clustered)	OR	95% CI for OR	p-value
(Intercept)	−0.612	0.094	0.542	(0.451, 0.652)	<0.001 ***
Temp anomaly (°C)	0.002	0.008	1.002	(0.987, 1.018)	0.788
Temp anomaly^2^	−0.016	0.014	0.984	(0.957, 1.012)	0.259
PAR (per unit, 0 → 1)	0.331	0.084	1.392	(1.180, 1.642)	<0.001 ***
PAR (per 1 SD, SD = 0.176)	0.058	0.015	1.060	(1.030, 1.091)	<0.001 ***
PAR (per 10 percentage-point increase)	0.033	0.008	1.034	(1.017, 1.051)	<0.001 ***
PAR × Temp anomaly	−0.015	0.018	0.985	(0.951, 1.020)	0.402
Energy expenditure (z)	−0.007	0.018	0.993	(0.957, 1.030)	0.694
[M] Mean Temp anomaly^2^ per prefecture	0.016	0.014	1.016	(0.988, 1.045)	0.259
[M] Mean energy expenditure per prefecture	0.007	0.018	1.007	(0.970, 1.045)	0.694
N (prefecture-years)	470				
McFadden pseudo-R^2^	0.002				
PAR average partial effect (proportion scale)	0.076				

Notes: β = fractional logit coefficient on the logit scale (Papke-Wooldridge 1996;19 quasi-binomial GLM with logit link; outcome SHR bounded in (0,1)). SE clustered at prefecture level (47 clusters). [M] = Mundlak correction term (group means of time-varying covariates only; the group mean of SHR itself was EXCLUDED, correcting the misspecification in the original submission). Year fixed effects included but not shown. PAR OR = exp(0.331) = 1.392; 95% CI: 1.180–1.642; average partial effect (APE) on the proportion scale = 0.076. McFadden pseudo-R^2^ = 0.002 (computed on the present fractional-logit specification; not directly comparable to the 0.966 reported in the originally submitted manuscript, which was an ordinary OLS R^2^ on a misspecified linear model that included SHR's own group mean as a regressor). *** p < 0.001; ** p < 0.01; * p < 0.05; † p < 0.10.

### 3.4 Counterfactual analysis for 2020

**Fig. 2:**
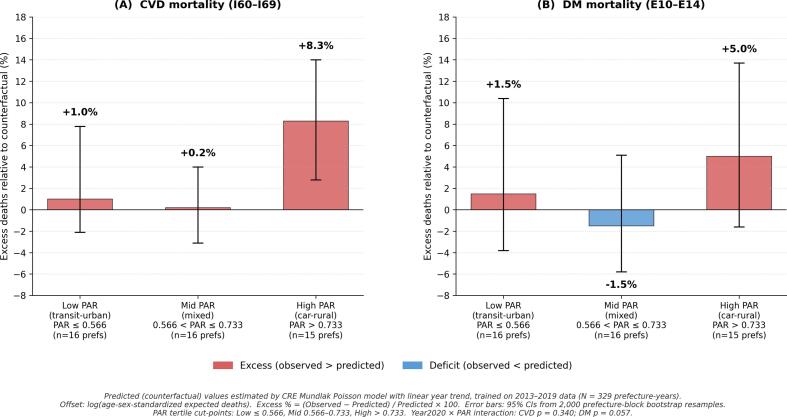
Counterfactual analysis of 2020 excess CVD and DM mortality by PAR tertile, with prefecture-block bootstrap 95% confidence intervals. Excess deaths (% relative to expected counterfactual values trained on 2013–2019 data) for CVD (cerebrovascular, I60–I69) and DM (diabetes, E10–E14) mortality, stratified by PAR tertile (Low: PAR ≤ 0.566; Mid: 0.566 < PAR ≤ 0.733; High: PAR > 0.733; equivalent to ≤56.6%, 56.6–73.3%, and > 73.3% car-only commute rates; *n* = 16, 16, 15 prefectures respectively). Error bars represent 95% bootstrap confidence intervals from 2000 prefecture-block resamples of the 2013–2019 training set. The High-PAR tertile showed +8.3% excess CVD deaths (95% CI +2.8% to +14.0%) and + 5.0% excess DM deaths (95% CI −1.6% to +13.7%). The corresponding continuous Year2020 × PAR interaction was not significant for CVD (*p* = 0.340) and borderline for DM (*p* = 0.057); see Discussion for interpretation of the apparent divergence between the tertile-stratified and continuous-PAR estimates.

Table 4 and Fig. 2 present the 2020 counterfactual analysis by PAR tertile, using the linear-trend specification trained on 2013–2019. Across all 47 prefectures, both CVD and DM 2020 deaths were close to their predicted counterfactual values at the national aggregate level (CVD: +1708 deaths, +1.7%; DM: +98 deaths, +0.7%), but stratification by PAR tertile revealed a consistent Low-to-High gradient.

**Table 4:** Counterfactual analysis 2020 — excess deaths by PAR tertile, with prefecture-block bootstrap 95% confidence intervals (linear trend trained on 2013–2019; 2000 bootstrap replicates).

Outcome	PAR Tertile (n prefectures)	PAR range	Observed 2020	Predicted (95% CI)	Excess count	Excess % (95% CI)
CVD (I60–I69)	Low PAR (transit-urban) (n = 16)	0.085–0.566	43,189	42,778 (40,063–44,101)	+411	+1.0% (−2.1% to +7.8%)
	Mid PAR (mixed) (n = 16)	0.566–0.733	43,760	43,682 (42,088–45,142)	+78	+0.2% (−3.1% to +4.0%)
	High PAR (car-rural) (n = 15)	0.733–0.790	15,951	14,732 (13,991–15,517)	+1219	+8.3% (+2.8% to +14.0%)
	All prefectures	0.085–0.790	102,900	101,192 (97,536–103,993)	+1708	+1.7% (−1.1% to +5.5%)
DM (E10–E14)	Low PAR (transit-urban) (n = 16)	0.085–0.566	6307	6215 (5712–6559)	+92	+1.5% (−3.8% to +10.4%)
	Mid PAR (mixed) (n = 16)	0.566–0.733	5639	5727 (5366–5986)	−88	−1.5% (−5.8% to +5.1%)
	High PAR (car-rural) (n = 15)	0.733–0.790	1950	1856 (1715–1983)	+94	+5.0% (−1.6% to +13.7%)
	All prefectures	0.085–0.790	13,896	13,798 (13,063–14,426)	+98	+0.7% (−3.7% to +6.4%)

Notes: Predicted (counterfactual) values estimated by CRE Mundlak Poisson model with linear calendar-year trend, trained on 2013–2019, and extrapolated to 2020 using observed 2020 covariates. Offset: log(age-sex-standardized expected deaths). Excess = Observed − Predicted; Excess % = Excess / Predicted × 100. 95% CIs from 2000 prefecture-block bootstrap resamples of the 2013–2019 training set, with model refit on each replicate. PAR tertile cut-points: Low (PAR ≤ 0.566; n = 16), Mid (0.566 < PAR ≤ 0.733; n = 16), High (PAR > 0.733; n = 15) — empirical tertiles of the 47-prefecture distribution. Robustness across alternative trend specifications (quadratic, piecewise, ARIMA) is reported in Supplementary eTable 5. The formal continuous Year2020 × PAR interaction test on the full 2013–2020 panel gave IRR = 1.030 (95% CI 0.969–1.096; p = 0.340) for CVD and IRR = 1.083 (95% CI 0.998–1.175; p = 0.057) for DM.

Cerebrovascular mortality showed a clear PAR gradient. The High-PAR tertile experienced +1219 excess CVD deaths (+8.3%; 95% CI: +2.8% to +14.0%), compared with +411 excess deaths in the Low-PAR tertile (+1.0%; 95% CI: −2.1% to +7.8%) and + 78 in the Mid-PAR tertile (+0.2%). The High-tertile CVD excess remained positive across all alternative trend specifications (linear +8.3%; quadratic +4.4%; piecewise +5.8%; Supplementary eTable 5). Diabetes mortality showed a directionally consistent but smaller gradient: High-PAR +94 excess deaths (+5.0%; 95% CI: −1.6% to +13.7%); Low-PAR +92 (+1.5%; 95% CI: −3.8% to +10.4%); Mid-PAR −88 (−1.5%). The formal continuous Year2020 × PAR interaction test gave IRR = 1.030 (95% CI: 0.969–1.096; *p* = 0.340) for CVD and IRR = 1.083 (95% CI: 0.998–1.175; *p* = 0.057) for DM, borderline significant for DM. These gradients are consistent with — but do not prove — the hypothesis that pre-existing structural PA constraints made high-PAR prefectures less able to absorb the 2020 disruption.

### 3.5 Sensitivity analysis

Within-prefecture FE specifications, in which PAR cannot be identified and was therefore dropped from the design matrix, yielded results for time-varying covariates consistent with the CRE Mundlak findings: temperature anomaly was not significantly associated with CVD or DM mortality, and energy expenditure remained positively associated with both outcomes (Supplementary eTable 2). Note that the previous draft reported a numerically non-zero PAR coefficient in the FE specification; this was a basis-dependent Moore–Penrose pseudoinverse artefact arising from rank deficiency (PAR is exactly collinear with prefecture dummies) and was not a valid statistical estimate. With PAR removed, the FE specification provides interpretable within-prefecture estimates of the time-varying covariates that corroborate the main model. Leave-one-prefecture-out (47 refits) and leave-one-year-out (10 refits) sensitivity analyses showed that the CVD-PAR coefficient was stable across all subsamples (IRR per SD min 1.059, max 1.084; CI lower bound >1 in 47/47 prefecture-LOO and 10/10 year-LOO refits; Supplementary eFigures S6–S7, eTables 7–8). Across hierarchical confounder models M0–M5 (Supplementary eTable 6, eFigure S8), the CVD-PAR coefficient was stable (IRR per SD 1.064–1.074), while the DM-PAR coefficient attenuated to non-significance after consumption-expenditure adjustment (M2 onward).

## 4 Discussion

### 4.1 Principal findings

This study set out to convert a first-principles claim — that the environments in which people live determine how much they walk, and therefore how their cardiovascular system is challenged across a lifetime — into an empirical effect-size benchmark for population health. Across all 47 Japanese prefectures over a decade, the proportion of commuters relying exclusively on cars — our index of structural physical-activity restriction (PAR) — was associated with greater cerebrovascular mortality, with greater diabetes mortality in unadjusted models, and with greater severe long-term-care need (SHR). The cerebrovascular signal persisted across all five hierarchical adjustment models (M0–M5: IRR per 1 SD ≈ 1.064–1.074, all *p* < 0.001) and across leave-one-prefecture-out and leave-one-year-out refits; across the observed PAR range, age-sex-standardized CVD mortality was approximately 29% higher in the most car-dependent prefecture (Yamagata) than in the least (Tokyo). The diabetes association attenuated to non-significance once household consumption expenditure was adjusted for, identifying car dependence in this respect as a marker of a correlated structural-disadvantage cluster (low income, low education, demographic ageing) rather than an isolated driver. The SHR association is the policy-relevant complement: structurally inactive prefectures not only died at higher rates from cerebrovascular disease but also accumulated a greater burden of severe long-term-care dependency, suggesting that the environmental PA constraint expresses itself in years lived dependent as well as in years of life lost — a distinction of central importance in a super-ageing society. The 2020 counterfactual analysis supplied the quantitative anchor: the High-PAR tertile recorded +8.3% excess CVD deaths (95% CI +2.8% to +14.0%) versus +1.0% in the Low-PAR tertile, alongside a directionally consistent but smaller DM gradient (High +5.0%, Low +1.5%). The formal continuous Year2020 × PAR interaction was not significant for CVD (*p* = 0.340) and only borderline for DM (*p* = 0.057); we read the tertile-stratified excess and the continuous-interaction IRR as complementary readings of the same data — the 2020 cardiovascular cost concentrated in the upper tail of the PAR distribution rather than scaling linearly with PAR across its full range. Together these results give a quantified, rather than presumed, environmental contribution to population cardiovascular mortality, and identify the populations in which an acute curtailment of behavioural PA most directly produced excess deaths.

### 4.2 Comparison with existing literature

The literature linking walkability and transit access to NCD outcomes at the population level is growing but predominantly from North America and Western Europe. Studies using Walk Score [9] and transit-access indices have consistently found lower rates of obesity, diabetes, and cardiovascular disease in walkable neighbourhoods [10], [11], and recent land-use/transport modelling work has estimated substantial NCD-prevention benefits of compact-city design [25]. Our findings extend this evidence to the Japanese context using an objective, census-derived structural measure rather than composite walkability scores, and show that the relationship operates at the prefecture level after controlling for multiple confounders through the CRE Mundlak estimator. The magnitude of our CVD-PAR association across the full observed range (IRR ≈ 1.29) is somewhat smaller than typical neighbourhood-walkability point estimates in Western settings but is in the same direction and broadly consistent with meta-analytic estimates of physical-inactivity → cardiovascular/diabetes risk at the individual level (RR ≈ 1.3–1.6),12 once ecological dilution is considered. Even after hierarchical adjustment, the CVD-PAR coefficient remains statistically meaningful, though it is likely best interpreted as a marker of a correlated cluster of structural characteristics of car-dependent prefectures — including but not limited to low ambient daily physical activity — rather than as an isolated independent causal driver of mortality.

The corrected Papke-Wooldridge fractional logit analysis of SHR yielded a positive PAR association (OR = 1.39, *p* < 0.001), reversing the null finding of the original misspecified linear OLS. The low fractional-logit McFadden pseudo-R^2^ (0.002) is the appropriate fit statistic for the corrected model and indicates that PAR and the other right-hand-side covariates together explain only a small fraction of the variation in SHR on the logit scale; the previous OLS R^2^ of 0.966 was a different statistic on a different (misspecified) model and reflected the mechanical inclusion of SHR's own group mean as a regressor, not genuine explanatory power. The positive PAR-SHR association suggests that, after correcting the analytical artefact, structural physical activity restriction is also associated with greater care severity, consistent with PA's role in preserving functional independence [23], [26]. Longer time series and mediation analyses would be needed to disentangle these mechanisms.

### 4.3 The COVID-19 counterfactual analysis

The 2020 PAR-tertile gradient in NCD excess mortality (CVD Low +1.0% vs High +8.3%; DM Low +1.5% vs High +5.0%) is, methodologically, what we asked the data to deliver: an effect size for an acute, society-wide curtailment of behavioural PA, contrasted across pre-existing structural PA environments. No deliberate experiment of this scale could ever have been performed for ethical reasons; the pandemic provided one. The pattern is what an environmental account would predict — populations with the least incidental PA to begin with, in car-dependent and low-walkability prefectures, had the least buffer to absorb the disruption — the empirical correlate of the ‘double disadvantage’ framing: pre-existing structural PA restriction and acute behavioural PA restriction compound rather than substitute. The absolute magnitude is modest, especially for DM (in the high-PAR tertile, +94 deaths over 1856 expected); the substantive value, however, lies in having a numbered estimate at all, against which the case for environmental intervention can be argued. A + 8.3% excess in the most car-dependent tertile is large enough that walkable-design and transit investment cannot be dismissed as marginal health policy; it is also small enough that residual confounding cannot be ruled out, and our analysis explicitly carries that caveat. Statistical precision is limited by the small ecological sample (15–16 prefectures per tertile), and many other 2020 changes — healthcare-seeking behaviour, cause-of-death certification practices, rural–urban differences in epidemic timing, dietary patterns, household economic stress, and telework adoption — could plausibly contribute to the gradient. The DM gradient is not strictly monotonic across tertiles (the Mid-PAR tertile showed −1.5%; 95% CI −5.8% to +5.1%), so we anchor our inferential claim to the continuous Year2020 × PAR interaction (DM *p* = 0.057, borderline; CVD *p* = 0.340, non-significant) rather than to tertile point estimates alone. The CVD tertile excess should be read as concentration of the 2020 shock in the most PA-restrictive prefectures, not as a linear PAR–excess relationship. Read alongside the long-run panel association, the wider implication is that environmental PA constraints accumulate a latent population vulnerability during ordinary times that is exposed when an acute mobility shock arrives — a finding with direct relevance for future pandemic preparedness as well as for ordinary-time urban-design policy.

### 4.4 Methodological contributions

This study contributes methodologically to ecological epidemiology in two ways. First, it demonstrates the application of the Mundlak (1978) Correlated Random Effects estimator to identify time-invariant structural exposures in ecological panel data, where within-prefecture fixed-effects estimators cannot. The Mundlak correction — adding group means of time-varying covariates — yields between-prefecture estimates of PAR conditional on the included time-varying covariates, under the conditional-mean independence assumption that the prefecture random effect, after this adjustment, is uncorrelated with PAR. It does not eliminate confounding by unobserved time-invariant prefecture characteristics. Second, the study combines this long-run panel model with a counterfactual-trend extrapolation for 2020, using prefecture-block bootstrap to quantify uncertainty in tertile excess percentages. Together, these provide two complementary, observational windows on the structural PA – mortality association, while remaining explicit that neither identifies a causal effect of PAR on mortality.

### 4.5 Policy implications

The findings give a quantitative answer to a question that walkable-cities and transit advocates have so far argued mostly on first principles: how much is the population-level cardiovascular mortality cost of car-dependent commuting environments? Our central estimate — a 6.4% higher CVD mortality rate per 1 SD of car-only commute share, a 29% gradient across the observed PAR range, and an acute +8.3% excess CVD mortality concentrated in the most car-dependent tertile during the 2020 PA shock — is large enough to be operationally meaningful and provides an empirical benchmark for health-economic cases that previously had none. The central message is therefore structural rather than behavioural: in environments where car dependence has displaced incidental walking from daily life, individual exhortations to exercise will struggle to compensate for the underlying environmental constraint, and the population burden of fatal and disabling NCDs will continue to track car dependence. First, investment in public transit, cycling infrastructure, and walkable urban design in high-PAR prefectures should therefore be framed as primary NCD prevention with a measured rather than presumed return, not solely as transport or environmental policy. The PAR–SHR association is particularly consequential here: structurally inactive environments produced not only excess cerebrovascular deaths but also greater accumulation of severe long-term-care need, meaning the years-lived-with-disability cost of car dependence falls on the long-term-care system as much as on mortality statistics. Second, even where structural redesign is slow, high-PAR prefectures warrant targeted public-health interventions (community exercise programmes, diabetes screening, fall and frailty prevention) calibrated to the reality of low incidental PA. Third, health-emergency preparedness should recognise that mobility-restricting measures will exert their largest marginal harms in communities whose ordinary-time PA infrastructure is already thinnest — the 2020 gradient is the empirical signal of this asymmetry, and a planning input rather than an after-the-fact observation for the next pandemic. We emphasise, however, that our findings are associational and that fully disentangling the structural cluster (PAR, income, education, age structure) would require richer prefecture-year data than currently available; the policy message does not depend on isolating PAR as an independent driver, because the cluster itself is what walkable-design and transit policy can simultaneously address.

## 5 Limitations

Several limitations should be noted. First, the design is ecological and subject to ecological fallacy [20], [21]: individual-level PA behaviours within prefectures may differ from the structural PAR measure, and individual-level inference is not warranted. Second, PAR is operationalised from the 2020 census and held constant across 2013–2022; while this is empirically defensible given strong structural persistence (rank correlation ρ > 0.97 between successive census rounds), it precludes modelling within-prefecture temporal change in PAR. Third, although hierarchical models M0–M5 added prefecture-year proxies for medical-facility density, income, education, age structure, and birth rate, several ideal confounders — individual smoking prevalence, dietary patterns, true physician counts, educational attainment ≥ tertiary, and time-varying ≥75 share — were not available at prefecture-year resolution in SSDSE-B and were substituted by proxies. Residual confounding therefore cannot be ruled out, and the PAR coefficient should be interpreted as a marker of car-dependent structural environments rather than an isolated causal driver. Fourth, the 10-year panel may be insufficient to detect long-latency CVD outcomes from PA behaviour changes; longer observation windows would strengthen inference. Fifth, the 2020 counterfactual analysis is a trend extrapolation rather than a formal interrupted time series or difference-in-differences design and does not satisfy parallel-trends or pre−/post-segmentation assumptions; many concurrent 2020 changes — healthcare-seeking behaviour, cause-of-death certification practices, rural–urban differences in epidemic timing and intensity, dietary changes, household economic stress, telework adoption — could contribute to the observed 2020 gradient and cannot be cleanly separated from the PA-disruption mechanism. Sixth, the small ecological sample (15–16 prefectures per tertile) limits statistical precision, particularly for the formal Year2020 × PAR interaction. Seventh, the outcomes are ICD-10 cause-specific mortality (I60–I69, E10–E14); we do not model mediation through metabolic risk factors, and the magnitudes should not be interpreted as deaths attributable to metabolic pathways.

## 6 Conclusions

Physical inactivity is conventionally framed as an individual-level risk factor; this study asks instead how much of the population-level cardiovascular mortality burden in Japan is attributable to the environments that make daily walking ordinary or impossible, and produces a numbered answer rather than an argument from first principles. Structurally car-dependent prefectures bore a higher burden of cerebrovascular mortality (approximately 29% across the observed PAR range, robust to age-sex standardization and to hierarchical adjustment for medical-resource density, income, education, and demographic structure) together with greater severe long-term-care need (SHR OR 1.39); the diabetes–PAR association attenuated upon socioeconomic adjustment, identifying car dependence in that respect as a marker of a correlated structural-disadvantage cluster rather than an isolated driver. The 2020 COVID-19 mobility shock — a society-wide curtailment of behavioural PA that could not have been imposed by design — provided a direct effect-size estimate: the most car-dependent tertile recorded +8.3% excess CVD deaths (95% CI +2.8% to +14.0%) above the pre-pandemic counterfactual trend, with essentially no excess elsewhere, identifying the populations in which acutely restricted PA most directly produced excess deaths. Methodologically, the study illustrates how the classical weakness of ecological designs — confounding — can be partly disciplined by stepwise hierarchical adjustment combined with Mundlak Correlated Random Effects identification of a time-invariant structural exposure: the asymmetric attenuation pattern (CVD–PAR robust across M0–M5, DM–PAR attenuating with consumption-expenditure adjustment) is itself an inferential signal that single-model analyses cannot deliver. Substantively, the effect sizes reported here are sufficient to argue that walkable urban design and accessible public transit should be treated as primary NCD prevention in super-ageing societies, not as ancillary transport or environmental policy, with the cost–benefit case for such investment now resting on measured rather than presumed population health gains. The design is ecological and the prefecture-level associations should not be interpreted causally for individuals; the message, however, is operational at the population level and identifies car-dependent prefectures as priority targets both for ongoing health monitoring and for protective planning during future mobility-restricting emergencies.

## Data sharing

The aggregated prefecture-year panel dataset used in this study is available from the corresponding author on reasonable request. All component data are publicly available from the sources described in the Methods.

## CRediT authorship contribution statement

**Akira Kimura:** Writing – review & editing, Writing – original draft, Visualization, Validation, Supervision, Software, Resources, Project administration, Methodology, Investigation, Formal analysis, Data curation, Conceptualization.

## Ethics statement

This study used only aggregated, publicly available administrative statistics at the prefecture level. No individually identifiable data were used. Ethics committee approval was therefore not required, in accordance with the Japanese Ministry of Health, Labour and Welfare's “Ethical Guidelines for Medical and Biological Research Involving Human Subjects” (revised 2022), which exempt research using only de-identified aggregate statistical data from prospective IRB review.

## Funding

This research received no specific grant from any funding agency in the public, commercial, or not-for-profit sectors.

## Declaration of competing interest

Declaration: I, Akira Kimura, declare that I have no conflicts of interest related to this manuscript. I have no financial or personal relationships with other people or organizations that could inappropriately influence this work.
