# Supplementary material for: Structural physical activity restriction, cerebrovascular and diabetes mortality, and long-term care intensity in Japan: An ecological panel study across 47 prefectures (2013−2022) with a COVID-19 counterfactual analysis
Source: Dialogues Health. 2026 Jun 11;9:100320. doi: 10.1016/j.dialog.2026.100320 (PMC13293738; doi:10.1016/j.dialog.2026.100320)
Supplement: Supplementary file 1 — Supplementary material 1 [file mmc1.docx]

# Supplementary Material

**Manuscript ID:** DIALOG-D-26-00141

**Title:** Structural Physical Activity Restriction, Cerebrovascular and Diabetes Mortality, and Long-Term Care Intensity in Japan: An Ecological Panel Study Across 47 Prefectures (2013–2022) with a COVID-19 Counterfactual Analysis

**Author:** Akira Kimura

## Contents

- eTable 1. Model dispersion parameters
- eTable 2. Fixed-effects sensitivity analysis (PAR dropped)
- eTable 3. SHR fractional logit (Papke-Wooldridge) full results
- eTable 4. Year2020 × PAR interaction test
- eTable 5. Counterfactual analysis — robustness across trend specifications
- eTable 6. Hierarchical confounder models M0–M5
- eTable 7. Leave-one-out (LOO) sensitivity summary
- eFigure S1. PAR distribution across 47 prefectures (existing, retained from original)
- eFigure S2. CVD and DM mortality time trends (existing, retained from original)
- eFigure S3. Temperature anomaly distribution (existing, retained from original)
- eFigure S4. PAR-CVD scatter (existing, retained from original)
- eFigure S5. PAR-DM scatter (existing, retained from original)
- eFigure S6. Leave-one-prefecture-out PAR coefficient stability (new)
- eFigure S7. Leave-one-year-out PAR coefficient stability (new)
- eFigure S8. Hierarchical confounder adjustment (M0-M5) forest plot (new)

## eTable 1. Model dispersion parameters

Pearson dispersion parameter (φ̂) for each Poisson outcome (calculated as Σ Pearson residuals² / residual df). Values > 1 indicate overdispersion relative to the Poisson assumption; cluster-robust standard errors absorb the additional variance.

| Outcome | Model | φ̂ (Pearson dispersion) | Interpretation |
| --- | --- | --- | --- |
| CVD (I60–I69) | CRE Mundlak Poisson with log(E_CVD) offset | 4.21 | Moderate overdispersion; cluster-robust SE used |
| DM (E10–E14) | CRE Mundlak Poisson with log(E_DM) offset | 2.86 | Mild overdispersion |
| T67 (Heat illness, validation) | CRE Mundlak Poisson with log(pop_elderly) offset | 6.13 | Larger overdispersion; sparse counts |

*Note. Values approximate; exact values depend on the dispersion estimator. Cluster-robust SEs absorb overdispersion-related variance.*

## eTable 2. Fixed-effects sensitivity analysis (PAR dropped, Reviewer 2 Comment #6)

Poisson regression with prefecture and year fixed effects, with PAR and PAR × Temp_anom DROPPED because they are exactly collinear with prefecture dummies. This sensitivity analysis confirms that the time-varying covariates (Temp_anom, Temp_anom², energy_z) behave as expected within prefectures.

| Outcome | Covariate | IRR | 95% CI | p-value |
| --- | --- | --- | --- | --- |
| CVD | Temp anomaly | 1.003 | (0.996, 1.009) | 0.397 |
| CVD | Temp anomaly² | 1.000 | (0.990, 1.010) | 0.968 |
| CVD | Energy expenditure (z) | 1.016 | (1.004, 1.028) | 0.010 |
| DM | Temp anomaly | 1.004 | (0.992, 1.017) | 0.479 |
| DM | Temp anomaly² | 1.008 | (0.991, 1.025) | 0.364 |
| DM | Energy expenditure (z) | 1.033 | (1.007, 1.060) | 0.013 |
| T67 (validation) | Temp anomaly | 1.580 | (1.438, 1.736) | <0.001 |
| T67 (validation) | Temp anomaly² | 1.043 | (0.927, 1.173) | 0.483 |
| T67 (validation) | Energy expenditure (z) | 0.920 | (0.791, 1.070) | 0.279 |

*Note. The strong temp_anom effect for T67 (heat illness) confirms the validation pathway. Energy expenditure (a proxy for residential AC use through utility costs) shows small but significant positive associations with CVD and DM.*

## eTable 3. SHR fractional logit (Papke-Wooldridge) full results (Reviewer 2 Comment #4)

Fractional logit (quasi-binomial GLM with logit link) for the severe care ratio (SHR = LTCI levels 3–5 / all certified). The Mundlak correction is applied ONLY to time-varying covariates (temp_anom², energy_z group means). The group mean of SHR itself is NOT included (correcting the original misspecification).

| Covariate | β (logit scale) | SE | OR | 95% CI for OR | p-value |
| --- | --- | --- | --- | --- | --- |
| (Intercept) | -0.612 | 0.094 | 0.542 | (0.451, 0.652) | <0.001 |
| Temp anomaly | 0.002 | 0.008 | 1.002 | (0.987, 1.018) | 0.788 |
| Temp anomaly² | -0.016 | 0.014 | 0.984 | (0.957, 1.012) | 0.259 |
| **PAR (per unit)** | **0.331** | **0.084** | **1.392** | **(1.180, 1.642)** | **<0.001** |
| PAR × Temp anomaly | -0.015 | 0.018 | 0.985 | (0.951, 1.020) | 0.402 |
| Energy expenditure (z) | -0.007 | 0.018 | 0.993 | (0.957, 1.030) | 0.694 |
| [M] Mean Temp anomaly² | 0.016 | 0.014 | 1.016 | (0.988, 1.045) | 0.259 |
| [M] Mean energy expenditure | 0.007 | 0.018 | 1.007 | (0.970, 1.045) | 0.694 |

**Summary statistics:** - N = 470 prefecture-years - McFadden pseudo-R² = 0.002 (the original 0.966 was an artifact of including SHR’s own group mean) - Average partial effect (APE) of PAR on the proportion scale = 0.076 (i.e., a 1-unit increase in PAR → SHR is 7.6 percentage points higher) - Year fixed effects included but not shown - SE clustered at prefecture level - PAR per 1 SD OR: 1.061 (per 0.176 increment)

## eTable 4. Year2020 × PAR interaction test (Reviewer 2 Comment #5)

Formal continuous-PAR interaction with the 2020 year dummy, replacing the informal tertile-gradient claim. Sample restricted to 2013–2020 (8 years).

| Outcome | β (interaction) | SE | IRR (Year2020 × PAR) | 95% CI | p-value |
| --- | --- | --- | --- | --- | --- |
| CVD (I60–I69) | 0.030 | 0.031 | 1.030 | (0.969, 1.096) | 0.340 |
| DM (E10–E14) | 0.079 | 0.041 | 1.083 | (0.998, 1.175) | **0.057** |

*Note. The DM interaction is borderline significant (p = 0.057), consistent with a stronger 2020 PA-restriction effect on DM in high-PAR prefectures. The CVD interaction is not significant at the continuous-PAR level, although the tertile-stratified counterfactual analysis (Table 4) shows a significant +8.3% excess in the highest tertile.*

## eTable 5. Counterfactual analysis — robustness across trend specifications (Editor Comment E9)

For each combination of (outcome, trend specification), excess deaths and excess percentages by PAR tertile are reported.

| Trend | Outcome | Tertile | Observed | Predicted | Excess | Excess % |
| --- | --- | --- | --- | --- | --- | --- |
| Linear | CVD | Low | 43,189 | 42,778 | +411 | +1.0% |
| Linear | CVD | Mid | 43,760 | 43,682 | +78 | +0.2% |
| Linear | CVD | **High** | 15,951 | 14,732 | **+1,219** | **+8.3%** |
| Linear | DM | Low | 6,307 | 6,215 | +92 | +1.5% |
| Linear | DM | Mid | 5,639 | 5,727 | -88 | -1.5% |
| Linear | DM | **High** | 1,950 | 1,856 | **+94** | **+5.0%** |
| Quadratic | CVD | Low | 43,189 | 44,416 | -1,227 | -2.8% |
| Quadratic | CVD | Mid | 43,760 | 45,366 | -1,606 | -3.5% |
| Quadratic | CVD | **High** | 15,951 | 15,285 | **+666** | **+4.4%** |
| Quadratic | DM | Low | 6,307 | 6,320 | -13 | -0.2% |
| Quadratic | DM | Mid | 5,639 | 5,824 | -185 | -3.2% |
| Quadratic | DM | **High** | 1,950 | 1,887 | **+63** | **+3.3%** |
| Piecewise | CVD | Low | 43,189 | 43,816 | -627 | -1.4% |
| Piecewise | CVD | Mid | 43,760 | 44,754 | -994 | -2.2% |
| Piecewise | CVD | **High** | 15,951 | 15,070 | **+881** | **+5.8%** |
| Piecewise | DM | Low | 6,307 | 6,266 | +41 | +0.6% |
| Piecewise | DM | Mid | 5,639 | 5,775 | -136 | -2.4% |
| Piecewise | DM | **High** | 1,950 | 1,871 | **+79** | **+4.2%** |

*Note. Bold rows: High-PAR tertile. The pattern of higher excess in the High-PAR tertile is consistent across all three trend specifications, supporting robustness of the counterfactual finding. The magnitude varies (CVD High: +4.4% to +8.3%; DM High: +3.3% to +5.0%) but direction is preserved.*

## eTable 6. Hierarchical confounder models M0–M5 (Reviewer 1 #3 / Reviewer 2 #2)

PAR IRR per 1 SD increase in each progressively-adjusted model. SSDSE-B proxy variables are used in place of the specific confounders requested (smoking, physician count, median income, education ≥tertiary, ≥75 share), which are not available at prefecture-year resolution.

| Model | Confounders Added | IRR per 1 SD (CVD) | 95% CI (CVD) | p (CVD) | IRR per 1 SD (DM) | 95% CI (DM) | p (DM) |
| --- | --- | --- | --- | --- | --- | --- | --- |
| M0 | Base (Temp, Temp², energy_z, Mundlak, year FE) | 1.064 | (1.037, 1.093) | <0.001 | 1.030 | (1.000, 1.060) | 0.049 |
| M1 | + Medical facility density (per 100k) | 1.065 | (1.039, 1.091) | <0.001 | 1.029 | (0.998, 1.061) | 0.067 |
| M2 | M1 + Consumption expenditure (log) | 1.074 | (1.051, 1.098) | <0.001 | **1.008** | (0.975, 1.042) | **0.650** |
| M3 | M2 + University student share | 1.069 | (1.039, 1.101) | <0.001 | 1.003 | (0.943, 1.067) | 0.920 |
| M4 | M3 + Age 65+ share | 1.066 | (1.028, 1.104) | <0.001 | 0.998 | (0.930, 1.072) | 0.963 |
| M5 | M4 + Birth rate (per 1000 pop) | 1.073 | (1.034, 1.114) | <0.001 | 1.011 | (0.937, 1.090) | 0.783 |

*Note. CVD-PAR remains highly significant (p < 0.001) across all five layers of adjustment, with IRR remaining at approximately 1.07. DM-PAR attenuates to non-significance upon adjustment for consumption expenditure (M2), consistent with DM-PAR capturing a marker of correlated structural socioeconomic conditions (low income, low education, demographic ageing) rather than an isolated independent driver. SSDSE-B confounders are used as proxies for the variables requested by Reviewer 2 (specifically: medical facility density ↔ physician count; log consumption ↔ median income; university student share ↔ education ≥tertiary; age 65+ ↔ age 75+). The limitations of these proxies are stated in the revised Methods and Discussion.*

## eTable 7. Leave-one-out (LOO) sensitivity summary (Editor Comment E10)

The model is refit 47 times excluding one prefecture at a time, and 10 times excluding one year at a time. The PAR coefficient (per 1 SD) and its CI are extracted at each iteration. A robust association should show IRR > 1 with CI_low > 1 across all LOO iterations.

### CVD (cerebrovascular mortality)

| Sensitivity | IRR per SD min | IRR per SD median | IRR per SD max | Iterations with CI_low > 1 |
| --- | --- | --- | --- | --- |
| Leave-one-prefecture-out (n = 47) | 1.059 | 1.065 | 1.084 | **47 / 47** |
| Leave-one-year-out (n = 10) | 1.062 | 1.065 | 1.070 | **10 / 10** |

### DM (diabetes mortality)

| Sensitivity | IRR per SD min | IRR per SD median | IRR per SD max | Iterations with CI_low > 1 |
| --- | --- | --- | --- | --- |
| Leave-one-prefecture-out (n = 47) | 1.018 | 1.030 | 1.036 | 22 / 47 |
| Leave-one-year-out (n = 10) | 1.027 | 1.030 | 1.032 | 7 / 10 |

*Note. CVD-PAR is fully stable (47/47 LOO-prefecture, 10/10 LOO-year), confirming that the result is not driven by any single prefecture (e.g., Tokyo as outlier with very low PAR, or Yamagata with very high PAR). DM-PAR is more marginal — 22/47 of LOO-prefecture and 7/10 of LOO-year iterations have CI_low > 1, consistent with the DM-PAR base-model association being weaker and more sensitive to specific prefectures, as also revealed by the M0–M5 hierarchical analysis.*

## eFigure Captions

### eFigure S1. PAR distribution across 47 prefectures

*(Retained from original submission, no changes.)* Bar chart of 2020 census PAR (car-only commute rate) for each of the 47 prefectures, sorted descending. Range: 8.5% (Tokyo) to 79.0% (Yamagata).

### eFigure S2. CVD and DM mortality time trends 2013–2022

*(Retained from original submission, no changes.)* National total mortality counts for CVD and DM from Vital Statistics of Japan, demonstrating the long-term gradual decline interrupted by 2020.

### eFigure S3. Temperature anomaly distribution

*(Retained from original submission, no changes.)* Boxplot of within-prefecture Jul–Aug temperature anomaly (deviation from 10-year prefecture mean) by year.

### eFigure S4. PAR vs CVD mortality (scatter)

*(Retained from original submission, no changes.)* Scatter of prefecture-mean 2013–2022 CVD mortality (per 100,000 population aged ≥65) against 2020 census PAR, with linear regression line.

### eFigure S5. PAR vs DM mortality (scatter)

*(Retained from original submission, no changes.)* Equivalent scatter for DM mortality.

### eFigure S6. Leave-one-prefecture-out PAR coefficient stability (NEW)

Forest plot showing CVD PAR IRR (per 1 SD) and 95% CI from 47 separate model fits, each excluding one prefecture. All 47 estimates fall in the narrow range 1.059–1.084, all with CI_low > 1. Demonstrates that no single prefecture drives the CVD-PAR result.

### eFigure S7. Leave-one-year-out PAR coefficient stability (NEW)

Forest plot showing CVD PAR IRR (per 1 SD) and 95% CI from 10 separate model fits, each excluding one year. All 10 estimates fall in 1.062–1.070, all with CI_low > 1.

### eFigure S8. Hierarchical confounder forest plot (M0–M5) (NEW)

Forest plot of CVD PAR IRR (per 1 SD) and 95% CI across the 6 hierarchical models. Visualizes the robustness of the CVD-PAR association to progressive confounder adjustment, in contrast to the attenuation pattern observed for DM (also shown as separate panel).

*All numeric results in this Supplementary file are derived from analyses on the 470 prefecture-year panel (47 prefectures × 2013–2022) constructed from official Japanese government statistical sources. Full data and analytic code will be deposited in a public repository upon acceptance.*
