# Supplementary figures and images for: Structural physical activity restriction, cerebrovascular and diabetes mortality, and long-term care intensity in Japan: An ecological panel study across 47 prefectures (2013−2022) with a COVID-19 counterfactual analysis
Source: Dialogues Health. 2026 Jun 11;9:100320. doi: 10.1016/j.dialog.2026.100320 (PMC13293738; doi:10.1016/j.dialog.2026.100320)

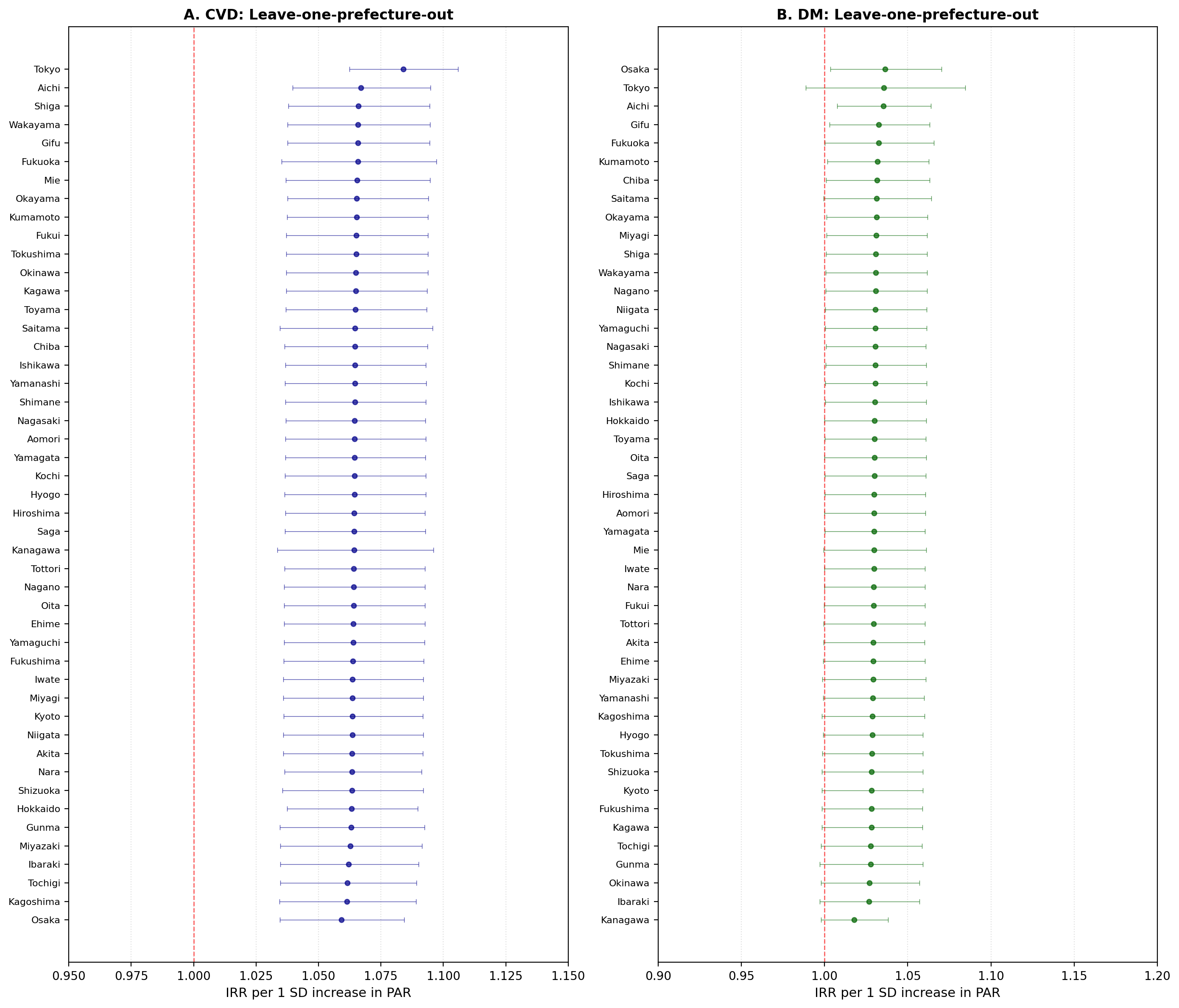

Supplement: Supplementary file 2 — Supplementary Fig. 1 [file mmc2.zip › eFigureS6_LOO_prefecture.png]

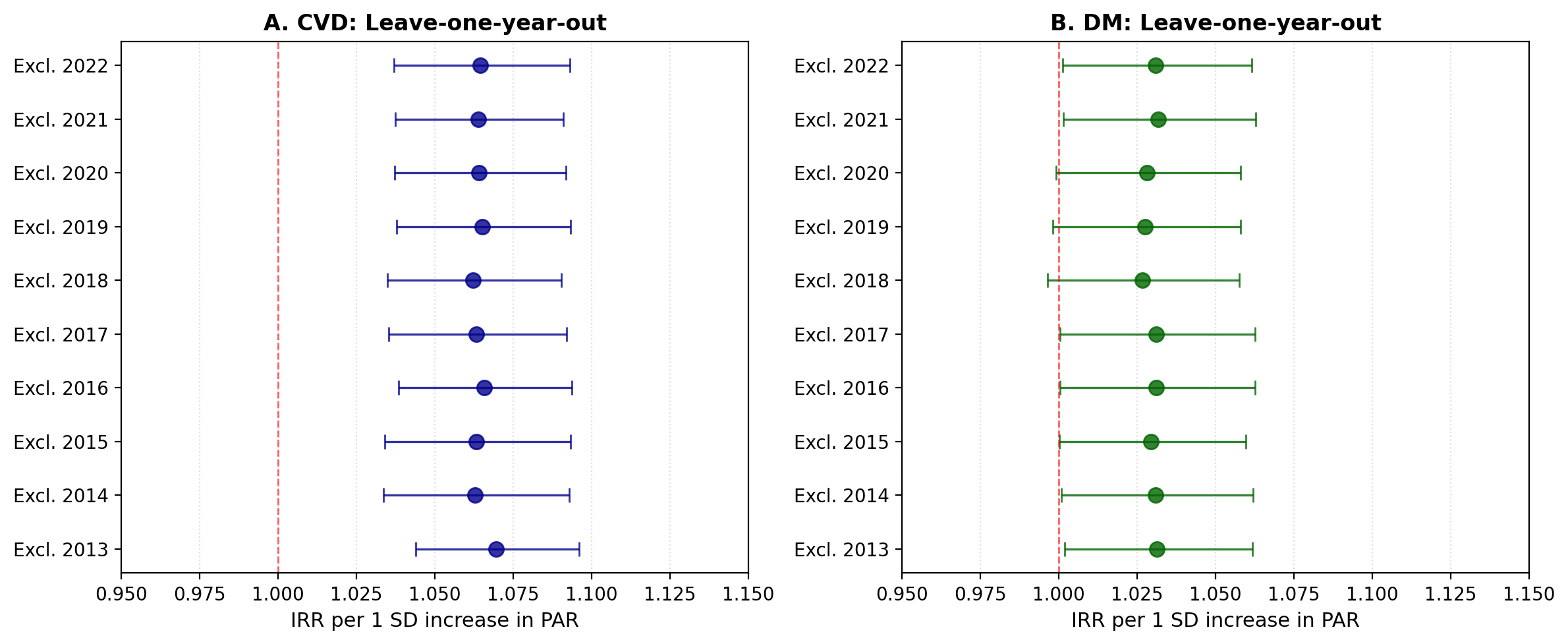

Supplement: Supplementary file 3 — Supplementary Fig. 2 [file mmc3.zip › eFigureS7_LOO_year.png]

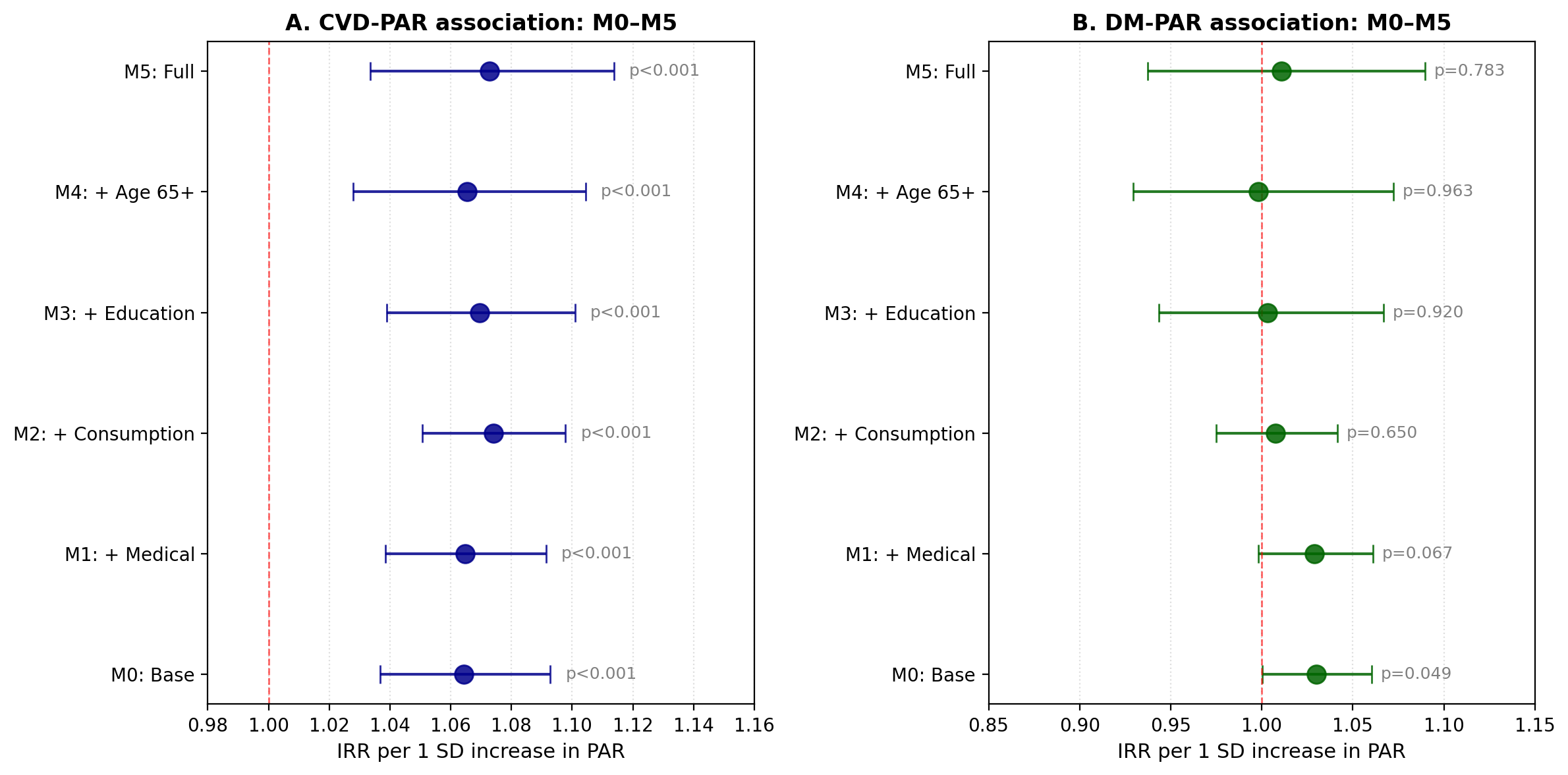

Supplement: Supplementary file 4 — Supplementary Fig. 3 [file mmc4.zip › eFigureS8_hierarchical_M0_M5.png]
